# Molecular Dynamics Simulation and Viscosity Analysis of Red Mud–Steel Slag Glass–Ceramics

**DOI:** 10.3390/ma16227200

**Published:** 2023-11-17

**Authors:** Wenjie Tan, Tao Sun, Fukun Ma, Min Jing, Liqiang Liu

**Affiliations:** School of Material Science and Engineering, Shandong Jianzhu University, Jinan 250022, China; tanwenjie19@sdjzu.edu.cn (W.T.); suntaosju@163.com (T.S.); mafukun18@sdjzu.edu.cn (F.M.); shandajingm@163.com (M.J.)

**Keywords:** molecular dynamics, glass–ceramics, viscosity, bond angle, red mud

## Abstract

The preparation of glass–ceramics with red mud and steel slag can not only solve the pollution problem caused by industrial waste slag but also produce economic benefits. It is difficult to analyze the high-temperature melt with the existing test methods, so the simulation experiment with molecular dynamics calculation becomes an important research method. The effects of steel slag content on the microstructure of red mud glass–ceramics were studied by molecular dynamics method. The results show that the binding ability of Si-O, Al-O, and Fe-O decreases with the increase in steel slag content. The number of Si-O-Si bridge oxygen increased gradually, while the number of Al-O-Al, Al-O-Fe, and Fe-O-Fe bridge oxygen decreased significantly. The number of tetrahedrons [SiO_4_] increased, the number of tetrahedrons [FeO_4_] and [AlO_4_] decreased, and the total number of three tetrahedrons decreased. The mean square displacement value of Si^4+^ and O^2−^ increases first and then decreases, resulting in the viscosity of the system decreasing first and then increasing. The molecular dynamics method is used to analyze the structure of red mud–steel slag glass–ceramics on the microscopic scale, which can better understand the role of steel slag and has guiding significance for the experiment of this kind of glass–ceramics.

## 1. Introduction

With the continuous improvement of computational materials science theory and the continuous improvement of computer computing speed, molecular dynamics methods can simulate more elements and larger systems [1,2,3,4,5]. The molecular dynamics method is not limited by time, space, and environmental conditions, which can reduce the danger and trouble of experiments and reduce the consumption of raw materials generated by experiments. It has become a powerful tool for the study of new materials of multi-component glass–ceramics [6,7,8]. With the enhancement of people’s awareness of environmental protection, glass–ceramics prepared from solid waste have become a research hotspot of high-value utilization of solid waste [9,10,11,12,13]. As the main components of red mud contain Na_2_O, CaO, Al_2_O_3_, and SiO_2_, commonly used in microcrystal systems, many researchers hope to prepare glass–ceramics from pure red mud. All attempts failed [14,15,16,17]. Therefore, in order to successfully prepare red mud glass–ceramics, other components must be added for regulation.

From the perspectives of maximum consumption of solid waste and reduced raw material cost, adding another solid waste can achieve the purpose of the regulation, which is the ideal method at present [18,19,20,21,22,23]. In this paper, the steel slag produced by steel mills is selected as the control material, and the influence of different steel slag additions on the red mud glass–ceramics system is discussed by molecular dynamics calculation and simulation method. The feasibility of preparing red mud glass–ceramics controlled by steel slag is analyzed from the microstructure and viscosity characteristics of the system.

## 2. Setting of Molecular Dynamics Simulation Parameters for Red Mud–Steel Slag Glass–Ceramics

Red mud and steel slag are the bulk solid waste produced by the metallurgical industry; the composition is more complex, and the main components are Na_2_O, CaO, Fe_2_O_3_, Al_2_O_3_, and SiO_2_, five oxides that contain some trace elements. Because the content of trace elements is very small, the influence of trace elements is not considered in this paper. The main mass percentage components of red mud and steel slag are shown in Table 1:

The initial model is introduced in the form of atoms. The number of simulated particles is about 2000, and the number of atoms depends on the proportion of oxides in the red mud glass–ceramics system. The choice of a 2000-particle system was made after a comprehensive consideration of computational accuracy and efficiency. Based on our computational experience, results tend to exhibit reduced accuracy when using fewer than 2000 particles. Conversely, beyond 2000 atoms, the computational outcomes show minimal variation (<5%), but the computational time experiences a substantial increase. In order to ensure the consumption of red mud, the content of additives is set to not exceed 50%, and the additional amount of steel slag is designed to be 0–50%. A total of six groups of different mass percentage composition ratios are simulated. The oxides of each group are shown in Table 2.

Materials studio 2017 software was selected for calculation simulation. The GULP module was used for microstructure calculation, and the Forcite module was used to analyze statistical data. In this paper, Materials Studio software is used for molecular dynamics simulation calculation. The potential function is very important for generating real structures and describing glass properties. Therefore, a modified Buckingham potential is chosen in this study, which is in the form of

$$U_{\left(r\right)}=\frac{z_{i}z_{j}{\mathrm{e}}^{2}}{r}+D_{ij}\left[{\left\{1-e^{-a_{ij}\cdot \left(r-r_{0}\right)}\right\}}^{2}-1\right]+\frac{C_{ij}}{r^{12}}$$

where $z_{i}$ and $z_{j}$ are the charges of the atom, and $r_{0}$ is the equilibrium bond distance. *D_ij_*, *a_ij_*, $r_{0}$ should simply be considered parameters.

The cooling mechanism of the red mud–steel slag glass–ceramics system is simulated by using the step-by-step cooling method, and the initial model runs 20 ps at 6000 K. Then, under the NVT ensemble, it is reduced to 2000 K at a speed of 1 × 10^14^ K/s with a duration of 40 ps. Then, 2000 K relaxation 20 ps is maintained in the NVE ensemble. After that, the temperature is cooled to 1800 K by running 20 ps under NVT, and the cooling rate is 1 × 10^13^ K/s. Then, 40 ps is run under the NVE ensemble to make the system reach the equilibrium state. The integral step was selected as 1fs, and the stable configuration was obtained and analyzed by molecular dynamics simulation.

**Figure 1:**
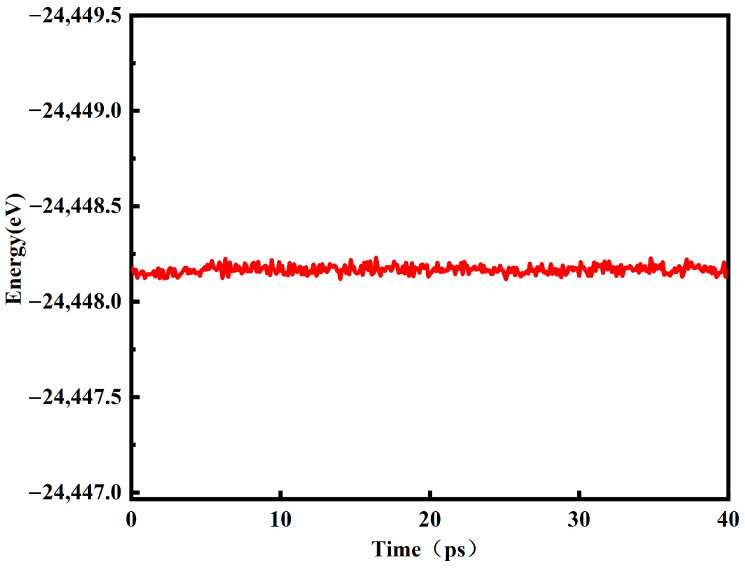
Energy variation diagram of red mud–steel slag glass–ceramics.

Figure 1 is the energy change diagram of the red mud–steel slag glass–ceramics system model running at 40 ps at 1800 K. It can be seen from the figure that the energy of the system is stable between −24,114 eV and −24,113 eV, and the amplitude of energy change is very small after 5 ps. According to the energy change, it can be seen that the molecular dynamics model of red mud–steel slag glass–ceramics is in a stable state at this time, so 40 ps is fully in line with the simulation conditions.

## 3. Results and Discussion

### 3.1. Bond Length Analysis

**Figure 2:**
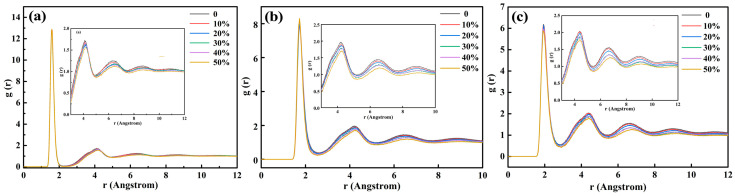
Radial function distribution of Si-O (**a**), Al-O (**b**), and Fe-O (**c**) of red mud-based glass–ceramics with different steel slag additions BI.

Figure 2a, Figure 2b, and Figure 2c are radial function distributions of Si-O, Al-O, and Fe-O under different steel slag contents, respectively. Si particles, Al particles, and Fe particles participate in the construction of the network, forming the structural unit; the first peak of the Si-O radial function is 1.60 Å, the first peak of Al-O radial function is 1.73 Å, and the first peak of Fe-O radial function is 1.95 Å, which is consistent with the actual situation. Compared with the radial distribution function between Al-O and Fe-O particles, the distribution range of Si-O particles is smaller, and the first peak position near the Y-axis is sharper, indicating that the binding ability between Si-O structures is stronger, and the network structure formed by Si-O particles is more stable. It can be seen that the radial distribution function of Si particles has two peaks, and that of Al particles and Fe particles has three peaks, which indicates that Si-O, Al-O, and Fe-O have multiple structures. With the increase in steel slag content, the second and third peaks of the radial distribution function of Si-O, Al-O, and Fe-O decrease, which indicates that the number of large bond lengths decreases, the average bond energy increases, and the binding force of the network structure increases.

### 3.2. Bond Angle Analysis

**Figure 3:**
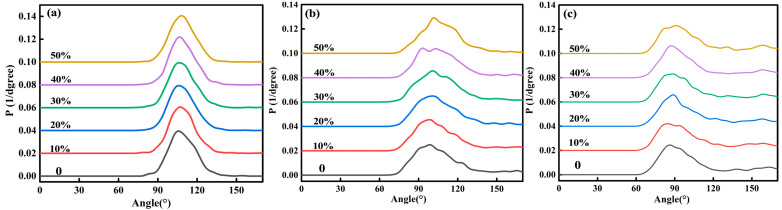
Distribution of O-Si-O (**a**), O-Al-O (**b**), and O-Fe-O (**c**) bond angles of red mud-based glass–ceramics under different steel slag contents.

Figure 3a, Figure 3b, and Figure 3c, respectively, show the distribution of O-Si-O, O-Al-O, and O-Fe-O bond angles in the red mud steel slag glass–ceramics system. It can be seen that the distribution law of bond angles is similar to the bond length, and the distribution range of O-Si-O bond angles is the smallest, while that of O-Fe-O is the largest. Among them, O-Si-O is mainly distributed between 90° and 120°. This range is basically consistent with the O-Si-O bond angle values of the compounds in the literature, such as 102.96°–115.05°, 101.91°–113.21°, and 109.56°–111.29° [22,23,24]. O-Al-O is mainly distributed between 75° and 125°, and O-Fe-O is mainly distributed between 70° and 130°, and both O-Al-O and O-Fe-O have weak peaks near 170°, indicating that Al-O and Fe-O have different structural units. By analyzing the structure of the reported compounds, it is found that the bond angles of O-Al-O are mostly in the range of 82.64°–119.61° [25,26,27], the bond angles of O-Fe-O are mostly in the range of 84.19°–121.04° [28,29,30], and a few of them are in the range of 173.04°–180.00° [26,29]. This shows that the calculation results can be basically consistent with the experimental data. With the change of steel slag content, the O-Si-O bond angle changes little, the O-Al-O and O-Fe-O bond angle peaks are affected, and the weak peaks of O-Al-O at 170° gradually disappear. 

### 3.3. Coordination Number Analysis

**Figure 4:**
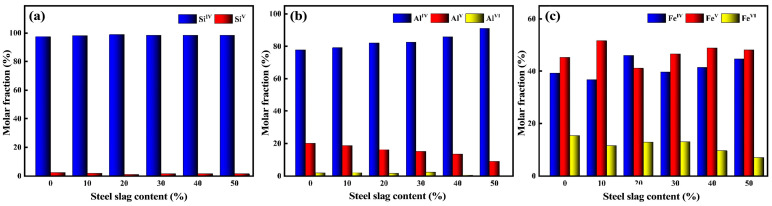
The proportion of Si-O (**a**), Al-O (**b**), and Fe-O (**c**) coordination numbers of red mud-based glass–ceramics under different steel slag contents.

The coordination number refers to the number of other atoms around the central atom of the compound within a specified radius [31]. It is an important parameter to characterize the structure of the model, and it can represent the condensation state and stability of the polymer. In this paper, the microstructure of glass–ceramics is simulated by molecular dynamics, and the number of different coordination numbers of each particle is obtained (Figure 4).

Figure 4a, Figure 4b, and Figure 4c are the coordination percentage content ratios of Si-O, Al-O, and Fe-O of red mud–steel slag glass–ceramics under different steel slag contents, respectively. It can be seen that in the molten state of red mud glass–ceramics at high temperatures, Si-O mainly exists in the form of four coordination numbers, and there is a small amount of five coordination numbers; the content of five coordination numbers is less than 3%. With the increase in the content of steel slag, the four coordination numbers increased, and the five coordination numbers decreased. Stebbins discovered the existence of five coordination numbers structure of Si in CaSi_2_O_5_ glass [32]. Lee et al. studied the glass transition temperature and structural properties of SiO_2_ at different temperatures through molecular dynamics simulation and observed the presence of a small amount of five coordination numbers in Si-O, indicating that there are five coordination numbers in Si in glass systems under some special circumstances [33]. 

It can be seen from Figure 4b that the coordination number of Al-O is relatively complex, with four coordination, five coordination, and six coordination. With the increase in steel slag content, Al-O four coordination gradually increases, while five coordination and six coordination gradually decrease. When the content of steel slag reaches 40%, the number of six ligands of Al-O is 0. The five coordination numbers and six coordination numbers of Al-O account for about 20% of the total number of Al-O ligands, which is much higher than the percentage content of the Si-O four coordination numbers, indicating that Al-O structural units are less stable than Si-O structural units. Fe-O and Al-O are similar in that they coexist in the form of four, five, and six ligands, but the content of five and six coo. The main reason for this change is that the coordination numbers of Fe-O are higher, indicating that Fe^3+^ is more likely to form highly coordinated structural units than Al^3+^ and Si^4+^. With the increase in steel slag content, the number of Fe-O six coordination numbers gradually decreases. In summary, with the increase in steel slag content, the high ligands of Al^3+^, Si^4+^, and Fe^3+^ are reduced, while the number of stable low ligands is increased, and the network structure in the system is more stable.

### 3.4. Analysis of Bridge Oxygen Number

Bridging oxygen refers to oxygen ions connected to two network tetrahedrons in a glass network structure; both the quantity and type of bridge oxygen can affect the stability and tightness of the network structure [34].

**Figure 5:**
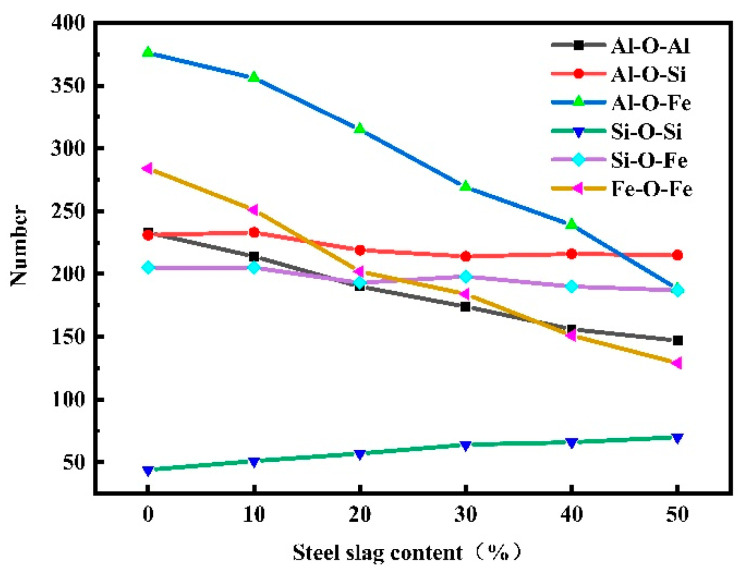
Variation of bridge oxygen quantity of red mud-based glass–ceramics under different steel slag content.

Figure 5 shows the distribution of bridge oxygen quantity of red mud–steel slag glass–ceramics under different steel slag content. In the red mud–steel slag glass–ceramics system, the number of bridged oxygen containing Al is the highest, which is more than half of the total bridged oxygen; the number of bridged oxygen containing Fe is the second; and the number of bridged oxygen containing Si is the least. With the increase in steel slag content, the Si-O-Si bridging oxygen quantity gradually increased, and the Al-O-Al, Al-O-Fe, Fe-O-Fe bridging oxygen quantity decreased obviously, while the Al-O-Si and Si-O-Fe bridging oxygen quantity showed a decreasing trend, but the decreasing quantity was very small. The main reason for this change is that after adding steel slag, the number of Fe and Al particles in the system decreases, and the number of Si particles increases, resulting in the number of particles participating in the construction of the network also changing. On the other hand, the average coordination number in the system is reduced, resulting in fewer O particles connected by Al and Fe and, ultimately, reducing the number of bridging oxygen.

### 3.5. Tetrahedral Number Analysis

**Figure 6:**
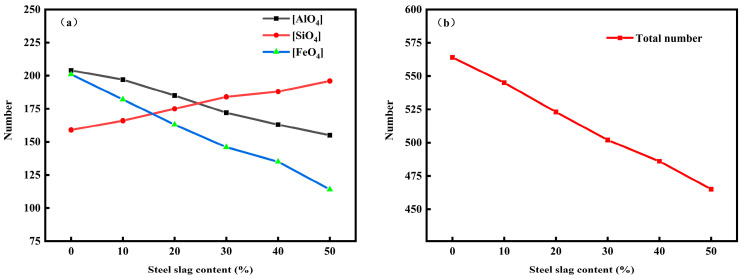
The number of [SiO_4_], [FeO_4_], [AlO_4_] tetrahedrons (**a**) and total number of tetrahedron (**b**) under different steel slag content.

Through molecular dynamics simulation of the microstructure of red mud-based glass–ceramics, it is found that the microstructure of red mud-based glass–ceramics is mainly composed of silicate network structure and floating ions floating between the networks, and the network structure formation is mainly in the form of tetrahedrons, including [AlO_4_], [SiO_4_], and [FeO_4_]. The number and type of tetrahedrons determine the stability and compactness of the network structure to a certain extent and have a major influence on the viscosity characteristics of the system. After molecular dynamics simulation of six groups of red mud–steel slag glass–ceramics, statistical analysis of the microstructure was performed to obtain the number variations of [AlO_4_], [SiO_4_], and [FeO_4_], as shown in Figure 6.

As can be seen from Figure 6, with the increasing of steel slag content, the number of [SiO_4_] tetrahedrons in the system continues to increase, and when the added amount of steel slag reaches 20% and 30% respectively, the number of [SiO_4_] tetrahedrons exceeds the number of [FeO_4_] and [AlO_4_] tetrahedrons, respectively. The number of tetrahedrons [FeO_4_] and [AlO_4_] has been decreasing with the increasing amount of steel slag. The main reason is that with the addition of steel slag, the content of SiO_2_ in the system gradually increases, and Al_2_O_3_ and Fe_2_O_3_ are decreasing, resulting in changes in the number of tetrahedrons in the system. According to the above results, the stability of [SiO_4_] is better than that of [AlO_4_] and [FeO_4_] in the three tetrahedrons of [AlO_4_], [SiO_4_], and [FeO_4_] in the microstructure of the network, so the network structure becomes more stable with the increase in steel slag. However, when the amount of steel slag added exceeds 30%, the tetrahedron growth rate of [SiO_4_] slows down, and the tetrahedron number reduction rate of [AlO_4_] and [FeO_4_] accelerates, indicating that after excessive steel slag added, the content of Ca^2+^ in the system increases, which plays a certain destructive role in the network structure. It can be seen that in the preparation of red mud–steel slag glass–ceramics, adding too much steel slag will reduce the mesh formation rate of the system, reduce the total number of tetrahedral bodies, and ultimately reduce the density of the network structure.

### 3.6. Analysis of Mean Square Displacement

**Figure 7:**
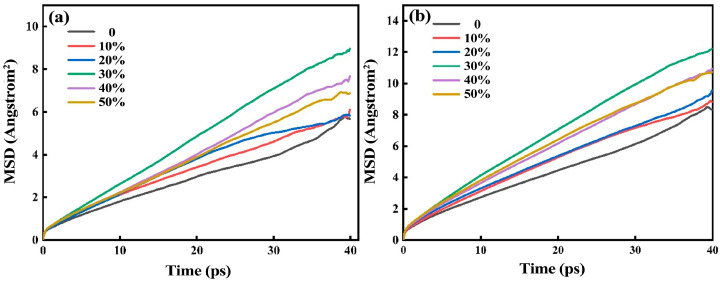
Mean square displacement of Si^4+^ (**a**) and O^2−^ (**b**) of red mud-based glass–ceramics under different steel slag content.

Figure 7 shows the distribution of the mean square displacement of particles of red mud-based glass–ceramics under different steel slag content. Mean square displacement refers to the distance of particle movement during a period of time in the simulation process, reflecting the movement rate of particles in the system. Through the movement rate of particles, the motion ability and viscosity of particles in the network structure can be deduced.

Figure 7a,b show the distribution of the mean square displacement of Si^4+^ and O^2−^ in the red mud–steel slag glass–ceramics system, respectively. It can be seen that the mean square displacement of Si^4+^ and O^2−^ changes in the same trend. With the increase in steel slag content, the mean azimuth shift increases first and then decreases, and there is a maximum value when the steel slag content is 30%. Because the mean square displacement can reflect the size of viscosity to a certain extent, it is speculated that there is an extreme value of viscosity change.

### 3.7. Viscosity Analysis

**Figure 8:**
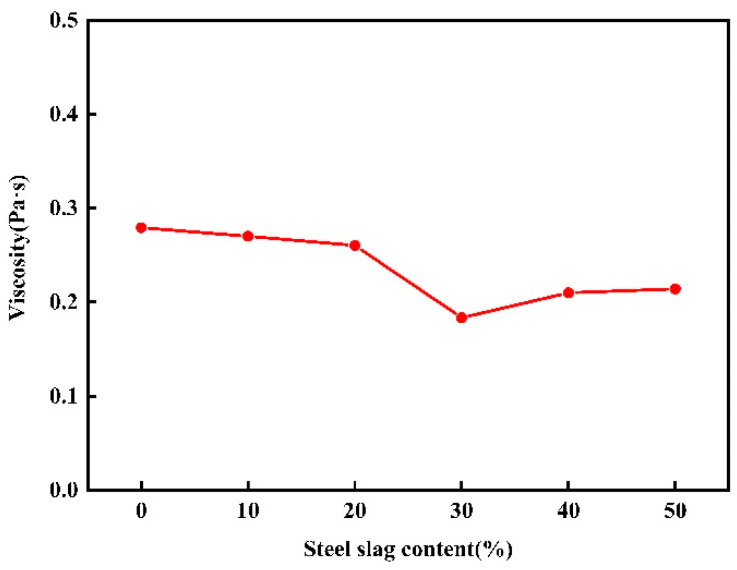
Viscosity variation of red mud-based glass–ceramics under different steel slag content.

The viscosity of glass–ceramics has a significant impact on various processes such as melting, forming, curing, heat treatment mechanism, and post-processing of glass. Additionally, it also influences the crystallization and mechanical strength of glass–ceramics. Viscosity analysis can be used to infer the structural state of the glass melt and to determine the casting and forming methods for glass–ceramics. In this paper, the viscosity changes in different components of red mud–steel slag glass–ceramics at 1800 K were calculated, and the results are shown in Figure 8.

It can be found from the figure that with the increase in steel slag content, the change of viscosity of red mud–steel slag glass–ceramics is not monotonous, first decreasing and then increasing, and there is a minimum value. It shows that the viscosity of molten glass–ceramics is closely related to its microstructure. With the increase in the content of steel slag, the tetrahedron content in the system gradually decreases, and the viscosity begins to decrease. When the steel slag continues to increase, [SiO_4_] in the system continues to increase and begins to occupy the dominant position of the network structure, and the unstable high ligand gradually decreases or even disappears, and the stable tetrad ligand gradually increases, resulting in the network structure of the glass becoming more stable, and the viscosity of the system begins to increase.

## 4. Conclusions

In this paper, six groups of microstructural models of red mud–steel slag glass–ceramics with different steel slag content were constructed, and the bond length, bond angle, coordination number, mean azimuth-shift, and viscosity of the system were calculated by molecular dynamics simulation. With the increase in steel slag content, the influence on the O-Si-O bond angle is small, and the influence on O-Al-O and O-Fe-O bond angle is large; the proportion of the four-coordination number of Si-O, Fe-O, and Al-O increased, while the number of higher coordination number decreased, the network structure in the system becomes more stable. When the steel slag content rises, the mean azimuth shifts values of Si^4+^ and O^2−^ first increase and then decrease, and there is a maximum value when the steel slag content is 30%, and the viscosity of red mud–steel slag glass–ceramics also decreases first and then increases. By comparing the effect of steel slag on the microstructure of glass–ceramics, it is found that the best content of steel slag in raw materials is 30%, and the products should have the best performance.

## Figures and Tables

**Table 1:** Composition table of red mud included in molecular dynamics simulation.

Materials	Na_2_O	CaO	Al_2_O_3_	SiO_2_	Fe_2_O_3_
Red mud (%)	12	8	25	20	35
Steel slag (%)	0	50	13	30	7

**Table 2:** Composition of different initial models of red mud–steel slag glass–ceramics (%).

Groups	Red Mud	Steel Slag	Na_2_O	CaO	Al_2_O_3_	SiO_2_	Fe_2_O_3_
1	100	0	12	8	25	20	35
2	90	10	10.8	12.2	23.8	21	32.2
3	80	20	9.6	16.4	22.6	22	29.4
4	70	30	8.4	20.6	21.4	23	26.6
5	60	40	7.3	24.8	20.2	24	23.8
6	50	50	6.1	29	19	25	21

## Data Availability

Data are contained within the article.
